# Machine learning based adaptive traffic prediction and control using edge impulse platform

**DOI:** 10.1038/s41598-025-00762-4

**Published:** 2025-05-17

**Authors:** Manoj Tolani, G. E. Saathwik, Ayush Roy, L. A. Ameeth, Dhanush Bharadwaj Rao, Ambar Bajpai, Arun Balodi

**Affiliations:** 1 Department of Information and Communication Technology, Manipal Institute of Technology, Manipal Academy of Higher Education, Manipal, Karnataka 576104 India; 2 Department of Electronics and Communication Engineering, Atria Institute of Technology, Bengaluru, Karnataka India; 3 Department of Electrical Electronics and Communication Engineering, GITAM Deemed University, Bengaluru, Karnataka India; 4 Department of Electronics and Communication Engineering, Dayananda Sagar University, Bengaluru, Karnataka India

**Keywords:** Traffic prediction, Traffic control, Artificial intelligence, Machine learning, TinyML, Internet of things, Electrical and electronic engineering, Computer science

## Abstract

Traffic congestion and delays are two major challenges in modern vehicle traffic control systems. These issues can be mitigated through an efficient and autonomous traffic scheduling system. The objective of the proposed methodology is to automate the traffic control system based on the density of vehicles approaching to the traffic signal without any human intervention. Unlike the conventional traffic signal systems that rely on preset timers which is often unsuitable for unpredictable traffic conditions. Therefore, the proposed approach dynamically adjusts signal timings based on real-time data. The methodology utilizes proximity sensors strategically placed at a predetermined distance from the traffic signal to detect approaching vehicles. The speed and density of vehicles are monitored based on the readings from these sensors. A Edge-Impulse-based machine learning model is proposed to predict the density and arrival time of the vehicles to the traffic signal. Using machine learning algorithms, the system can forecast future traffic conditions and optimize real-time traffic control by significantly reducing congestion and delays. Moreover, by automating the traffic scheduling process, the proposed methodology can help to reduce human error and improve the safety of road users. The proposed methodology has the potential to transform existing traffic control systems, making them more intelligent, efficient, and autonomous. The model is rigorously tested and validated to ensure its reliability and accuracy in real-world traffic scenarios.

## Introduction

India, with 16% of the world’s population, is the second most populous nation and has a rapidly expanding economy in various sectors. With a length of 5.6 million km, the nation has one of the greatest road networks in the world, facilitating approximately 64% of all commodities traffic and 90% of passenger travel. However, the cost of transportation, which is crucial for the nation’s development, has a direct impact on the environment, businesses, and consumer health. Traffic congestion has grown to be a serious issue in today’s 5G environment that negatively impacts our everyday life. Recent advancements, such as multi-level intelligent control systems integrating inter-vehicle communication between smart traffic lights and autonomous vehicles^1^, have demonstrated the potential of intelligent coordination in traffic management. The drone-assisted cooperative D2D communications for traffic monitoring in 5G networks^2^ have further highlighted the role of emerging technologies in reducing congestion and improving overall traffic efficiency.

For instance, the Indian metropolis of Delhi experiences severe traffic congestion, leading to increased travel time and air pollution. Although the legal speed limit in Delhi ranges from 40 to 55 km/h, the average traffic speed remains around 26 km/h, rising only slightly to 27 km/h during non-peak hours. In addition, there is barely any distinction between peak and off-peak times. Advanced technologies, such as the Green-Tech CAV framework, which integrates traffic sign and obstacle detection in connected and autonomous vehicles, offer promising solutions to improve traffic flow and reduce congestion in urban environments like Delhi^3^. Long-term traffic exposure might cause health issues like myocardial infarction. Therefore, it is essential to raise traffic signal efficiency in order to address these problems^4^.

The three primary lights in India’s current traffic control system (TCS) are red, green, and orange, which stand for stop, start, and get ready to start/stop, respectively. Currently, traffic lights are positioned in various locations with fixed time delays, following a specific cycle, and switching from one signal to another while maintaining the other lanes, which causes unneeded congestion on one lane^5^. But in addition to the growing number of vehicles on the road, more traffic lights have made the contra issue worse. Due to traffic light timings that are predetermined and do not take into account the changing traffic density throughout the day, the current TCS is inefficient^6,7^. Adaptive traffic control systems, which incorporate dynamic signal timers and emergency vehicle prioritization, have demonstrated their potential to overcome inefficiencies associated with fixed signal timings. These systems play a crucial role in enhancing traffic flow within intelligent traffic management frameworks^5^.

Automation in areas like transit, defense, industrial automation, and smart homes has benefited greatly from machine learning (ML). Despite having a wealth of data to automate, the traffic algorithms remain static. Although the ideology of traffic automation may be gaining ground, there are still many real obstacles to overcome.

Induction loops, a monitoring technology that has been used since the 1960s to surmount these obstacles, is more recent than cameras that can track traffic and read license plates. However, the use of image and video processing to identify vehicles is susceptible to unfavorable weather conditions like fog and mist. Additionally, the range of these technologies is constrained by their expensive and high transmission bandwidth. The best way to optimize traffic signals in the twenty-first century is to use adaptive traffic management systems. They are made to be ready for anything because transportation patterns are not always consistent. Based on the present and anticipated traffic situation, adaptive traffic control systems are used to manage both recurring and non-recurring congestion dynamically. They place a lot of emphasis on trip reliability, maximize the facility’s effectiveness and efficiency, and boost throughput and safety by utilizing seamless systems and new technologies^8,9^.

The area of automation has benefited significantly from cloud computing. Recent research has focused on the drawbacks of cloud computing, including data privacy, network bandwidth, latency, reliability, and energy efficiency. The growth of cloud-based automation systems is hampered by a number of threats. Edge computing is used to address these problems. Tiny machine learning (TinyML), a developing edge computing idea, combines machine learning and embedded systems. TinyML enables smart choices to be made by devices without transferring data to the cloud, improving efficiency and privacy^10^.

Based on the above discussions, this article proposes an Edge-Impulse-based machine learning solution to predict green/red signal timings based on real-time traffic density analysis. The present work introduces a cost-effective and scalable solution leveraging TinyML and Edge Impulse Studio^11^ to address inefficiencies in current traffic control systems. The existing system has some limitations of static traffic signal algorithms, which fail to adapt to varying traffic densities, leading to congestion and delays. The proposed approach integrates real-time data collection using proximity sensors and employs a neural network-based machine learning model. The proposed system is designed primarily for internal urban networks, such as city junctions, where traffic density and vehicle flow patterns are dynamic and require adaptive control mechanisms. The system is specifically designed for managing traffic at signalized junctions, where accurate and timely decision-making can significantly reduce congestion and enhance traffic flow. The novelty and contribution of the proposed approach are outlined below:

- In the present work, we have integrated tinyML, Arduino Nano 33 BLE Sense, and Edge Impulse Studio^11^ to develop a highly accurate and cost-effective alternative to predict the adaptive traffic on signals in major cities.
- The paper proposes a novel approach for predicting and adapting traffic control using tinyML-based Neural Network Model.
- A prediction model is designed and developed using Edge Impulse Studio and Arduino IDE by considering 18 input features and seven output classes of traffic for the prediction of junction traffic.
- The proximity sensor of Arduino Nano 33 BLE Sense is used in the prototype to simulate real-time traffic scenarios to train the IoT network and to analyze the performance.
- The neural network-based Edge Impulse method is used with Tensorflow and Keras libraries to predict the traffic. More than 55000 data samples are collected from a prototype for the feature analysis and traffic classification.
- The performance is analyzed using a prototype model and small-scale experimental scenario to mimic the large-scale real-time traffic.

The paper’s organization is as follows: The related works are presented in Section II. In Section III, the modelling of the proposed traffic prediction model is thoroughly explained. Design and simulation are covered in Section IV. The analysis of the experimental findings is presented in Section V. The findings are concluded in Section VI.

## Related works

Sridevi et al. proposed a ML method for the traffic congestion detection based on multiple parameters, e.g., delay constraints and the speed of the vehicle^12^. The GPS trajectory model is used for the estimation of speed. The machine learning based gaussian process is used for predicting traffic speed and have used three datasets, i.e., training set, road sector data frame, and prediction set. Three different time slots are used for the vehicle traffic congestion monitoring and estimating the average velocity of the vehicles on the road sector during respective time slots. It is evident that the proposed approach has several advantages over traditional traffic monitoring techniques.

Xueyan et al. have conducted a comprehensive analysis of deep learning-based methods for traffic prediction from multiple perspectives^13^. The authors present a taxonomy and a summary of the traffic forecasting techniques currently in use. Additionally, the authors have listed the cutting-edge techniques used in various applications for traffic prediction and have thoroughly compiled and arranged widely-used public datasets from the literature, which can help other researchers working in this field.

Alfonso Navarro-Espinoza et al. introduced machine-learning and deep-learning algorithms for estimating traffic flow at an junction to create the framework for adaptive traffic control^14^. The proposed models were trained, validated, and tested using two open datasets, and the performance of different machine learning and deep learning techniques was contrasted. The best outcomes were obtained using the Multilayer Perceptron Neural Network (MLP-NN), demonstrating that smart traffic signal controllers might apply it. The study shows how machine learning and deep learning algorithms can be used to address issues like traffic congestion in the real world.

The researchers have reported many traffic control protocols, however, in the previously reported work, the researchers have not developed an experimental model for the performance analysis of real-time traffic^10,15–17^. In^18^, the authors have used a surveillance camera and reinforcement learning for efficient traffic control, which is a costlier solution and requires huge installation expenses. In^19,20^, the authors have used reinforcement learning-based optimization methods for the existing dataset to avoid the collision. In addition, in previously reported works, the researchers have used various ML-based conventional algorithms^21^. Conventional algorithms, like Random forest, Decision Tree, or KNN are suitable for models expecting data in the form of images or videos. They are expected to work more complicated and consume more time. The algorithm we are utilizing focuses on using the least amount of data being given, which is the value of proximity sensor output. This reduces the processing time drastically. Hence there is no requirement to use such algorithms that misinterpret the sensor data and produce less accuracy.

Qaddoura et al. developed optimized machine learning techniques for real-time traffic prediction using data from roads in multiple countries, enhancing prediction accuracy with metaheuristic algorithms and improving performance metrics like R-squared and RMSE^22^. Similarly, Elalouf et al. applied machine learning to predict the severity of pedestrian injuries in road accidents, emphasizing the model’s adaptability to large datasets and its potential for improving road safety by identifying critical risk factors^23^. Kim et al. introduced a hybrid framework combining simulation and machine learning to improve real-time traffic prediction, particularly under unforeseen events like crashes and adverse weather conditions, highlighting the benefits of combining model-based and data-driven approaches^24^. Harrou et al. focused on traffic flow prediction using deep learning and wavelet transforms to capture non-linear patterns in traffic data, achieving high prediction accuracy^25^.

Ma et al. proposed deep learning machines optimized method with swarm intelligence algorithms for traffic prediction and autonomous lane changes. It demonstrates superior results in both tasks^26^. Li et al. combine machine learning with a Bayesian spatial Poisson model to predict large-scale real-time traffic conflicts, emphasizing the need for separate predictions for conflict occurrence and frequency^27^. Berhanu et al. used machine learning and spatial network analysis to predict traffic accidents and suggest safer routes, improving traffic safety and reducing congestion^28^. Pérez Moreno et al. developed a machine learning model to predict air traffic complexity, helping optimize air traffic flow^29^. Lu et al. introduced a method for traffic flow prediction using decomposition and gated recurrent units, improving data accuracy^30^. Cicek et al. compared machine learning models for predicting traffic accident severity, focusing on explain-ability^31^, while Sufian et al. integrated machine learning with econometric techniques for road traffic accident severity prediction, leveraging Explainable AI (XAI) to identify key contributing factors^32^.

In previous studies, a rigorous analysis of early traffic prediction for a specific junction based on the traffic conditions at interconnected junctions has not been reported. In our current study, a traffic prediction system is created using machine learning techniques, which have proven efficient due to its traffic estimation based on real-time dataset. The proposed approach provides precise traffic prediction. The system’s performance is evaluated using a traffic generation prototype to make real-time predictions. To the best of our knowledge, a neural network-based dense intermediate layer and feature extraction method for real-time traffic models and real-time dataset has not been developed for vehicle traffic prediction and control. The proposed approach uses tensorflow library and powerful kerasflow optimizing models that can accurately classify the traffic with high precision. Our study has contributed to this field and highlights the potential for further development of real-time traffic prediction and control systems.

**Fig. 1:**
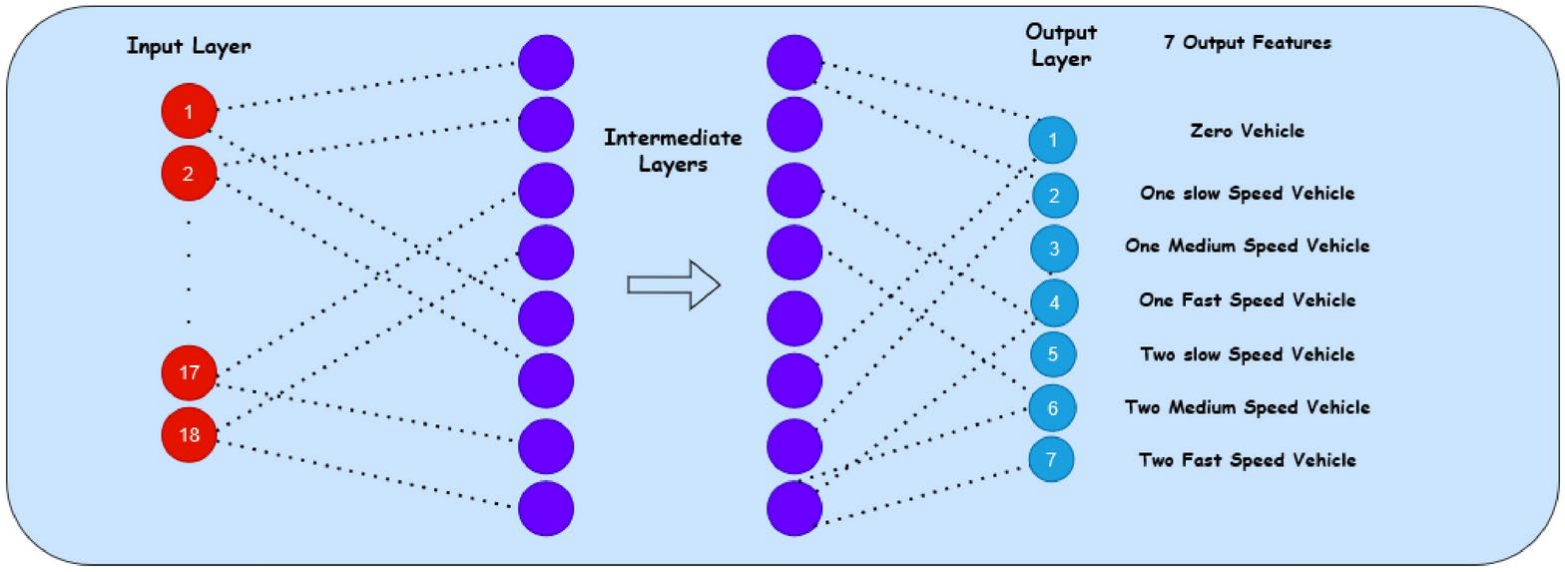
Proposed NN Model for Traffic Prediction. A schematic of the neural network architecture designed for predicting traffic patterns for different numbers of vehicles.

## Methodology and modelling

The proposed research addresses a significant gap in the existing literature on traffic signal control. While the literature primarily focuses on optimizing traffic signal timings through rule-based or reinforcement learning methods, the proposed approach introduces a novel dimension to traffic management. Unlike the traditional systems, the proposed methodology employs proximity sensors placed strategically at a distance from traffic signals/junctions to autonomously detect the number of vehicles and speed as they approach. Additionally, the Edge-Impulse-based machine learning model is integrated to predict both the speed of the vehicles and the arrival time of vehicles accurately. This combination of sensor technology and machine learning distinguishes our work from the predominantly rule-based approaches discussed in the literature. It allows real-time data-driven traffic control that is adaptable to dynamic traffic scenarios. We believe that this approach not only has the potential to significantly reduce congestion and delays but also enhances road safety by minimizing human intervention. The aim is to reduce the wait time and ideally provide no halting near signals for vehicles. Based on the prediction, signals can be controlled. Therefore, a prediction model is designed and developed using Edge Impulse Studio^11^ and Arduino IDE by considering 18 input features and seven output classes of traffic for the prediction of junction traffic. The neural network is shown in Fig. 1.

**Fig. 2:**
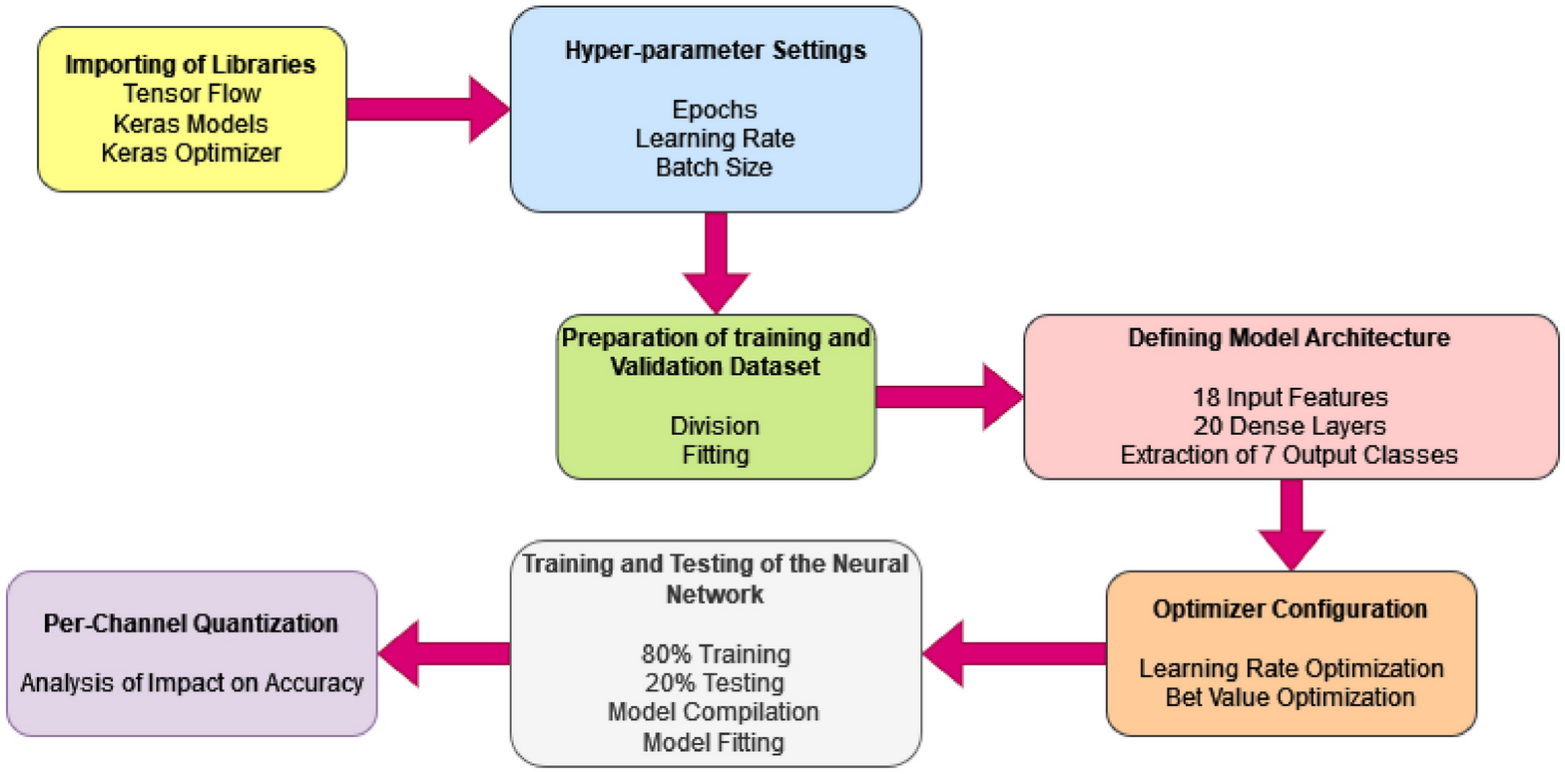
Methodology of the proposed model. Step-by-step approach detailing the process and techniques used in developing the traffic prediction model.

### Proposed algorithm

The process flow of the proposed method is explained in Algorithm 1 and shown in Fig. 2. The training process begins with the design of a neural network model structured to efficiently classify traffic density into seven distinct output classes: ’Zero-Vehicle,’ ’One Slow Speed Vehicle,’ ’One Medium Speed Vehicle,’ ’One Fast Speed Vehicle,’ ’Two Slow Speed Vehicle,’ ’Two Medium Speed Vehicle,’ and ’Two Fast Speed Vehicles.’ The neural network architecture, implemented using TensorFlow and Keras libraries, consists of three dense layers with specific activation-ReLU for the first two layers and Softmax for the output layer-optimally designed for classification tasks. These layers are tuned to process 18 input features, representing sensor data, to extract meaningful patterns relevant to traffic scenarios. Hyperparameter optimization, including a learning rate of 0.0005 and batch size of 32, ensures effective training. A batch logger callback is added to monitor training progress, and the model is trained over a specified number of epochs ($N = 1700$). During training, the algorithm provides an option to control per-channel quantization, which, if disabled, can reduce RAM usage at the potential cost of accuracy. The trained model is finally returned as the output.

**Algorithm 1:**
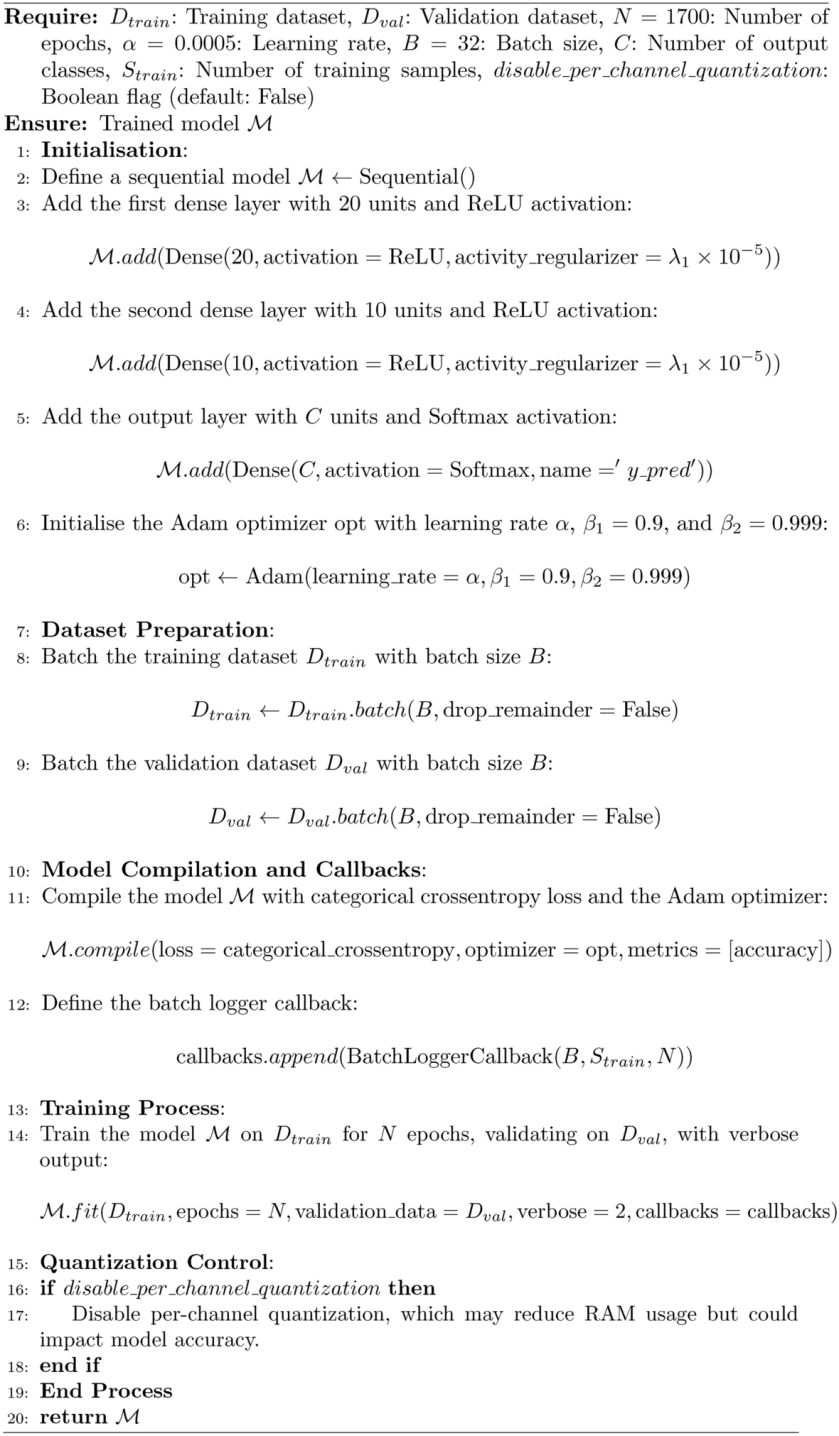
Training a Neural Network Model with TensorFlow/Keras

The algorithm starts with defining a sequential neural network model, comprising three dense layers. The first two dense layers have 20 and 10 units, respectively, each with ReLU activation and regularization to avoid overfitting. The output layer consists of 7 units, corresponding to the seven output classes. The Adam optimizer is initialized with a learning rate of $\alpha = 0.0005$, ensuring stable and efficient convergence during training.

**Table 1:** Hyperparameter Settings for the Proposed Model.

Parameter	Value
Training Cycles	1700
Learning Rate	0.0005
FFT Length	16
Processing Time	1 ms
Frequency of Time Series	50 Hz

**Table 2:** Data Acquisition and Labelling.

Sample	Label	Time Length
$zero\_3rd5349o$	zero	2s
$one\_slow3rd4ddedp$	$ones\_low$	2s
$one\_med3rdaffai$	$one\_med$	2s
$one\_fast3rdtyffi$	$one\_fast$	2s
$two\_slow3rd78gffd$	$two\_slow$	2s
$two\_med3rdxtyio$	$two\_med$	2s
$two\_fast3rdewqty$	$two\_fast$	2s

**Fig. 3:**
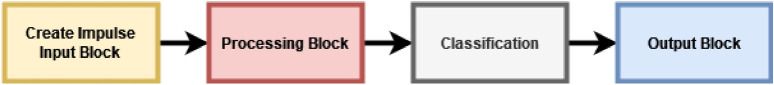
Impulse Modelling Steps. Illustration of the stages involved in impulse response modeling for signal processing.

The spike generation in the time samples helps in the estimation of the speed of the vehicles. The two-vehicle analysis is replicated for the highly dense traffic of multiple vehicles. The process steps used for the modeling are shown in Fig. 2. The process involves importing libraries, configuring hyperparameters, preparing datasets, and defining a neural network with 18 input features and 7 output classes. The model is trained (80%) and tested (20%), with optional per-channel quantization for optimizing edge-device performance. The tensor flow, Keras model, and keras optimizer libraries are used to analyze the proposed model. The hyperparameter settings are given in Table 1. The preprocessing methods are used for the preparation of training and validation of dataset. The neural network of 18 input features with 20 dense layer is used for the extraction of 7 output classes as shown in Fig. 1 and Table 2.

The extracted output is optimized with the help of various optimizers as shown in Fig. 2. Edge impulse Studio^11^ is used to train models for computer analysis with embedded machine learning. The platform chosen for its robust support for TinyML workflows, efficient model optimization, and seamless integration with real-world sensor data, aligning with the study’s objectives. To train a model, the data sets are split into two buckets, a training set used to train the algorithm and a test set used to ensure that the algorithm is working properly. The 80% data samples are used for the training, and 20% of the data samples are used for the testing. In the end, the accuracy of the proposed method is analyzed per-channel quantization. The detailed code is attached in the ’Supplementary Material’. In the proposed work, an Edge Impulse Studio platform is used for the modeling of the traffic prediction system. The process is divided into four steps, as shown in Fig. 3. The details of the steps are discussed in the below subsections.

### Create impulse

The time samples are taken to generate times series data. The time duration between successive data points in a time series is an important factor to consider when analyzing and forecasting time-based data. Input axes (6) red, green, blue, brightness, proximity, gesture. These features are measured by the onboard IMU sensor and are used as input to a machine-learning model.

A window size of 2000 ms time interval is taken to analyse the data. Longer time interval is taken due to the large collection of data. Window increase of 1000 ms is employed for smooth estimation. The frequency of 50 Hz is used which helps to determine the appropriate window size, assess the stability, and forecast accuracy of the model.

### Processing block

The processing blocks are divided into 2 sub-blocks, i.e., flatten and spectral analysis.

#### Flatten

**Fig. 4:**
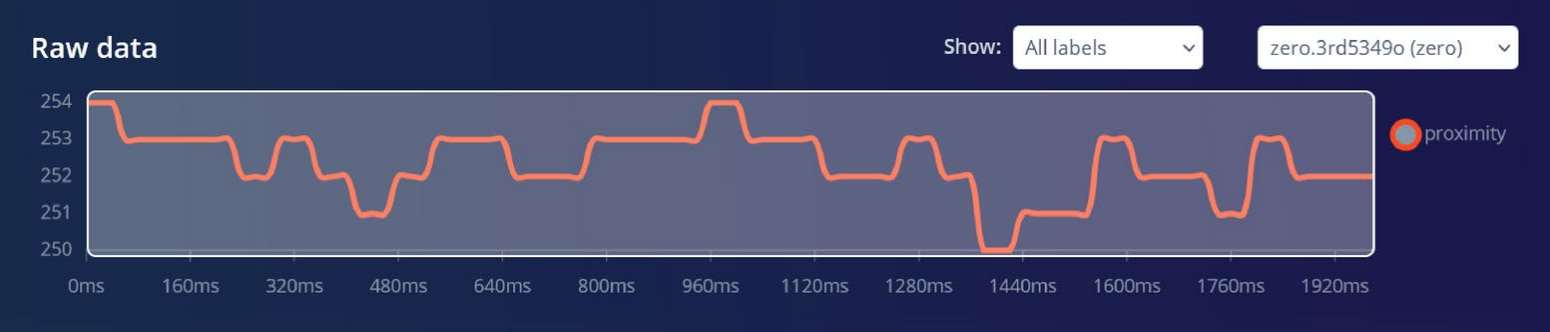
Raw Data time samples plot. Graph showing the initial, unprocessed time series data collected from traffic sensors.

**Fig. 5:**
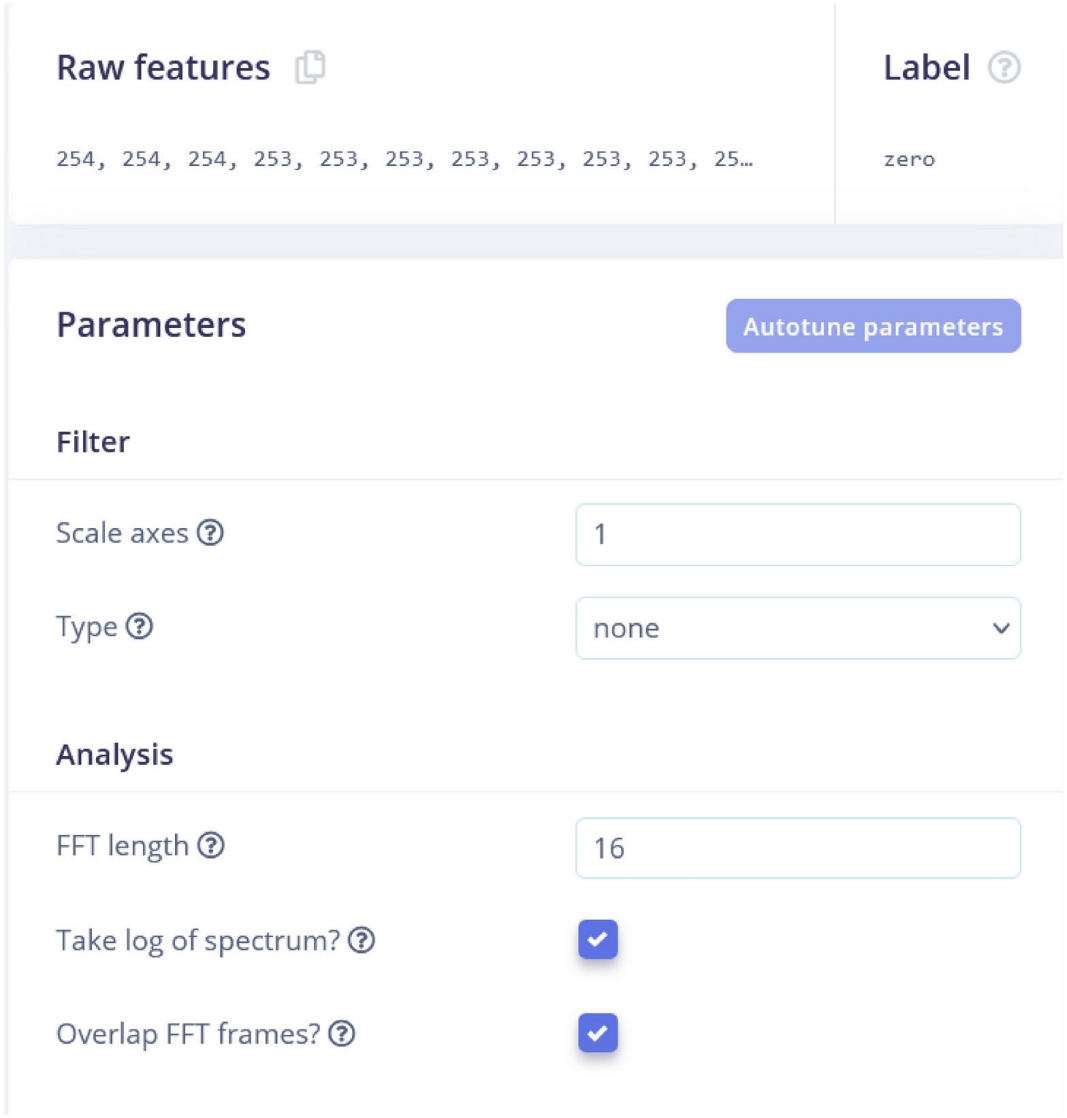
Feature values and Parameters. Visualization of the extracted features from the raw data, used as inputs for the model.

**Fig. 6:**
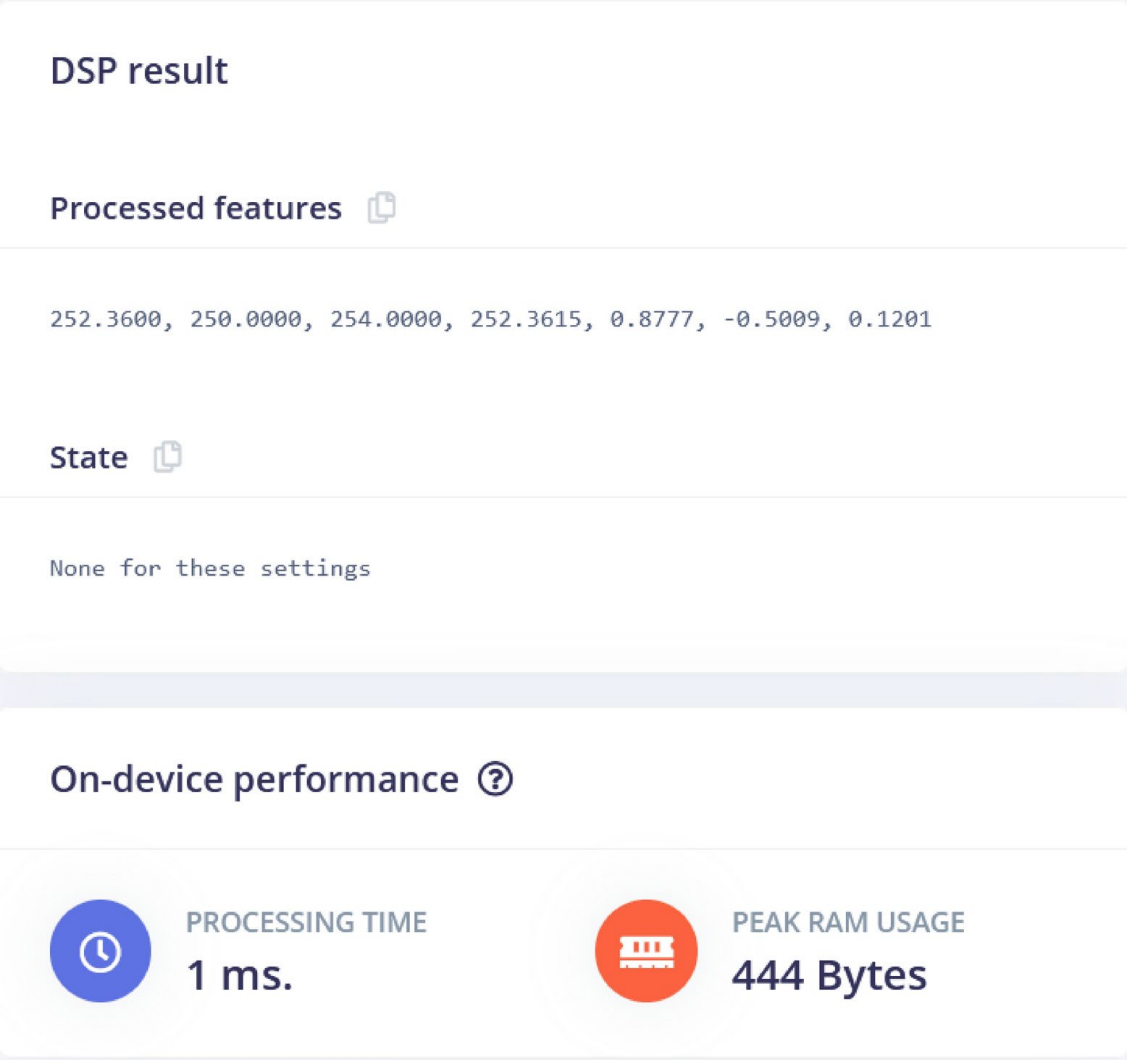
DSP Results. Outcomes of the digital signal processing techniques applied to the raw data.

**Fig. 7:**
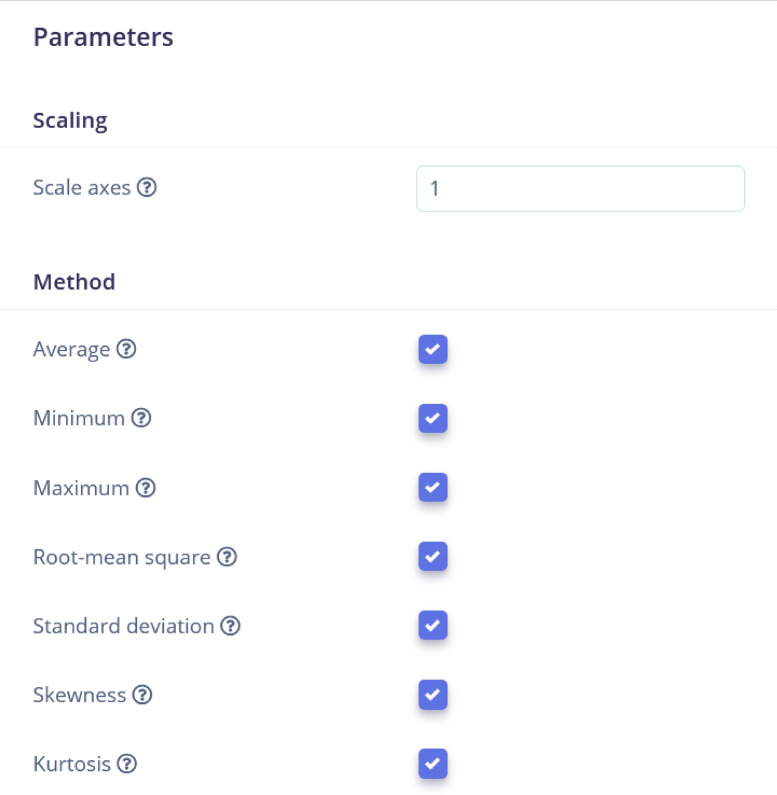
Analysis Parameters. All the important analysis parameters are given.

The Flatten processing block converts multidimensional data into a one-dimensional array, making it suitable for input into a machine learning model. Proximity is selected under flatten because it is a feature that can be extracted from the proximity sensor and can be used to train machine learning algorithms. Raw features are the input data collected from the sensor, while processed features are the output of the processing blocks that extract meaningful features from the raw data. Sample raw data, raw features and parameters, and DSP results are shown in Figs. 4, 5 and 6. Figure 4 shows a time series plot of proximity data over a duration of approximately 1920 milliseconds. The proximity values fluctuate between 250 and 254, indicating variations in distance or signal strength detected by a sensor. The graph highlights these fluctuations, which is presenting the changes in the environment or movement relative to the sensor. In Fig. 5, ”Raw features” section lists the collected data values (e.g., 254, 253, etc.), while the ”Parameters” section allows the user to set up filters and analysis methods. The ”Scale axes” is set to 1, and no filtering type is applied. For analysis, the FFT (Fast Fourier Transform) length is set to 16, with options to take the logarithm of the spectrum and overlap FFT frames both enabled. These settings is used so that the data will be analyzed in the frequency domain with specific preprocessing steps (Fig. 7).

Figure 6 shows ”State” section indicates that there are no specific states or conditions associated with the settings used. In terms of on-device performance, the processing time was 1 millisecond, and the operation required 444 bytes of peak RAM usage, reflecting efficient resource usage for the task.

#### Spectral analysis

**Fig. 8:**
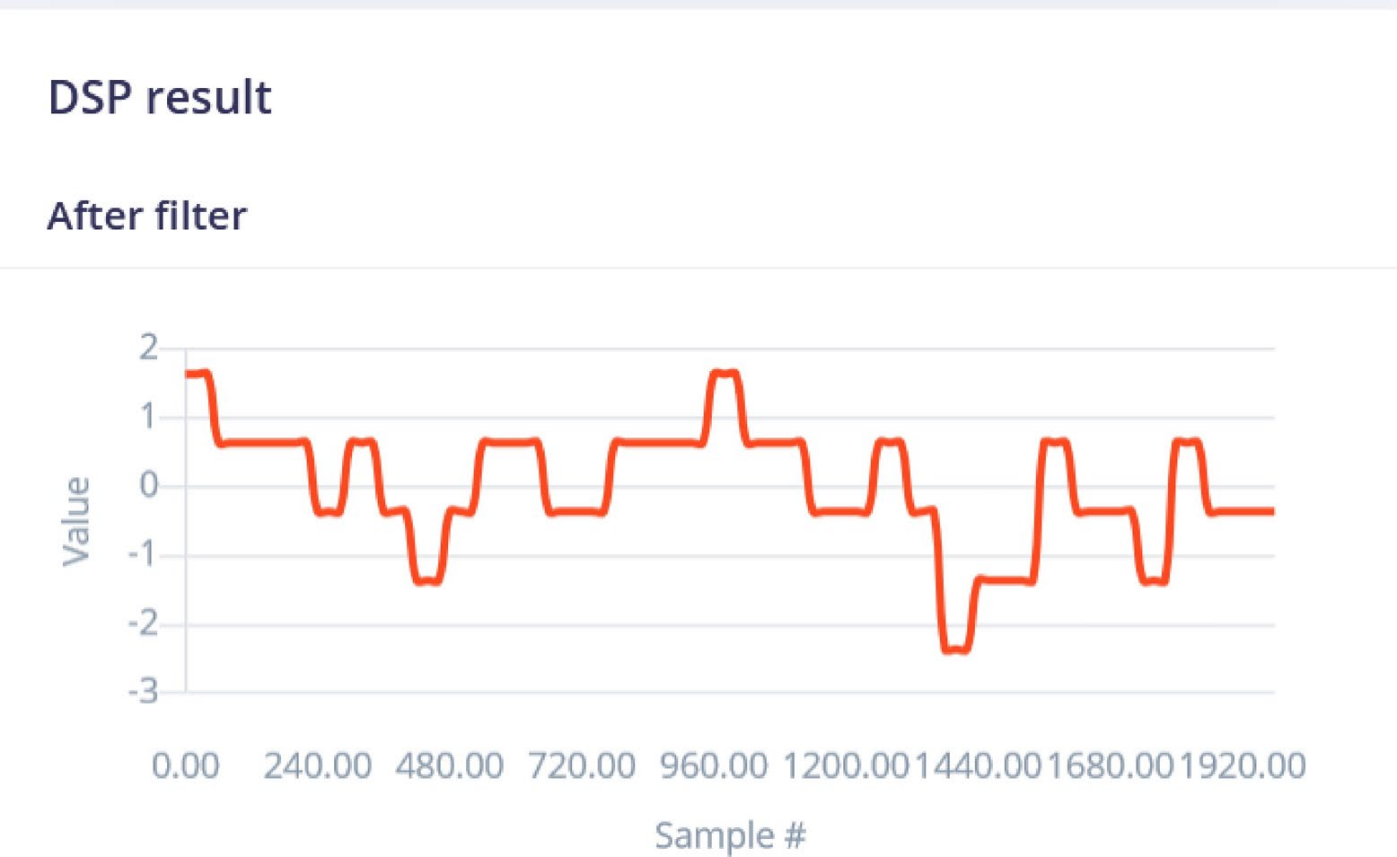
DSP Results after filtering. Filtered signal data showing the results post application of digital signal processing filters.

The spectral analysis processing block was used for repetitive patterns in the signal. The Spectral Analysis processing block in Edge Impulse is a feature extraction block that converts time-domain signals into frequency-domain signals. It does this by applying the Fourier transform to the input signal, which converts the signal from the time domain to the frequency domain. The resulting frequency-domain signal contains information about the frequency content of the input signal. The DSP results after filtering is shown in Fig. 8 for the raw data shown in Fig. 4. The analysis parameters are given in Fig. 7. The ”Scaling” parameter is set to’1’ to ensure the consistent data normalization, which is important for maintaining the integrity of comparisons across different data points. The selected methods-Average, Minimum, Maximum, Root-mean square, Standard deviation, Skewness, and Kurtosis-are statistical measures that capture essential characteristics of the data, such as central tendency, dispersion, and distribution shape. The selected metrics are important because they help in identifying patterns, trends, and anomalies, ultimately improving the accuracy and effectiveness of predictive models. The parameters are properly configured to ensure that the extracted features are representative and meaningful, leading to more reliable model performance.

**Fig. 9:**
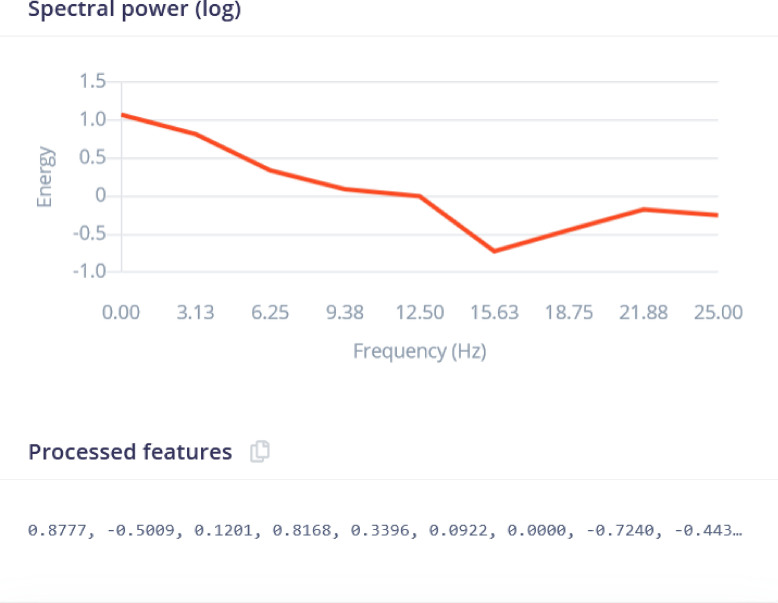
Spectral power logs. Logarithmic plot of the spectral power distribution (Energy) across different frequencies in the data.

The spectral diagram (powerlog) is also shown in Fig. 9 which indicates that the spectral power distribution across frequencies is concentrated at lower frequencies and decreases as the frequency increases, with a slight rise at higher frequencies. The processed features represent key characteristics of the signal’s frequency components, summarizing the signal’s behavior for further analysis or modeling tasks.

### Classification

**Fig. 10:**
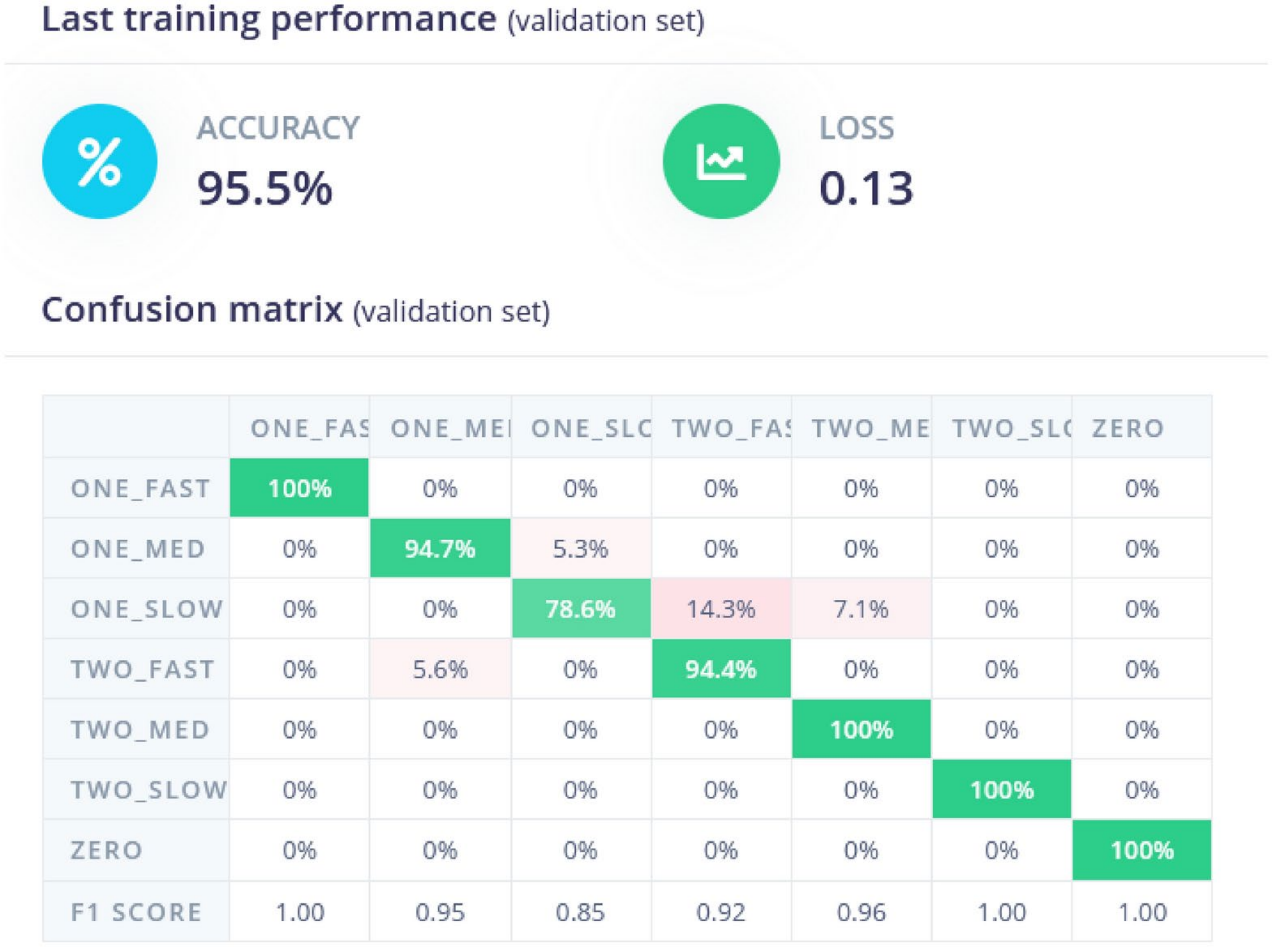
Classification of data samples. Categorization of data samples into various classes based on model predictions (zero vehicle, one vehicle, 2 vhicles, 2nd low, medium and fast speed).

The classification model is trained on a labeled dataset, where each data point is associated with a specific class or label, and the goal is to predict the class of new, unlabelled data points. The classification of samples is shown in Fig. 10. The training performance results indicate that the model achieved a high accuracy of 95.5% on the validation set with a low loss of 0.13, suggesting strong predictive performance. The confusion matrix shows that most of the predicted classes align well with the actual classes, especially for categories like ”ONE-FAST” and ”ZERO,” both with 100% accuracy. However, there are minor misclassifications in categories like ”ONE-SLOW,” which was misclassified as ”TWO-FAST” 14.3% of the time. The F1 scores for all categories are above 0.85, indicating a balanced performance between precision and recall across all classes.

### Output feature extraction block

This feature is extracted from the input data which is used to train the machine learning module. In machine learning, the output of a model can be a probability distribution, a vector of scores, or a class label. These outputs can be further processed to extract useful information that can be used for downstream tasks.

## Simulation and design

**Fig. 11:**
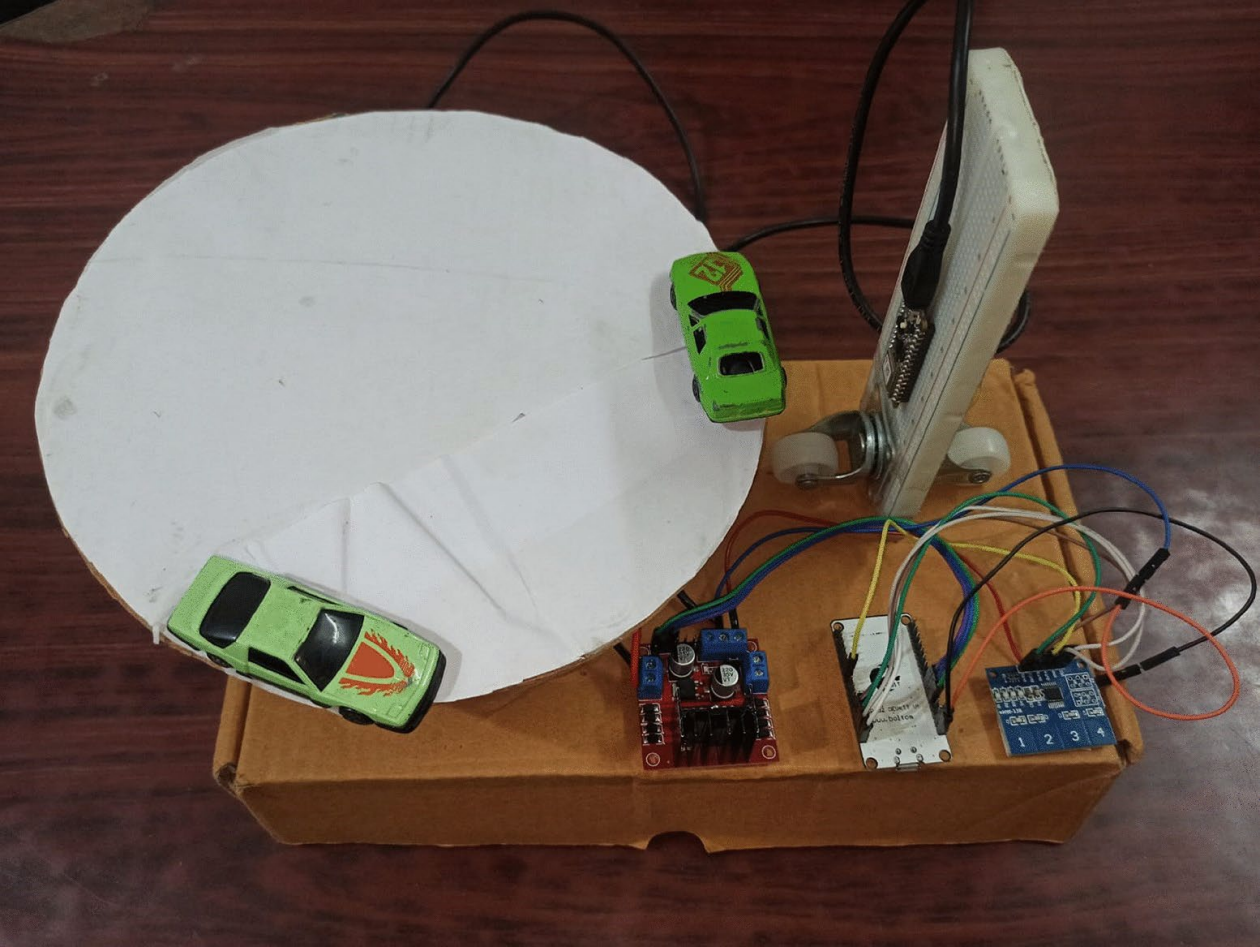
Prototype of the proposed method. Preliminary version of the model implementation showing the proposed workflow.

For the simulation analysis, the data-set is collected from the prototype. The prototype is shown in Fig. 11. The prototype is designed to collect the real-time traffic data and predict traffic conditions by using machine learning methods. It features a circular platform, representing the continuous linear movement of vehicles, with two toy cars simulating traffic flow. Sensors detect the cars’ speed, proximity, and movement on the platform. The main objective of the prototype is to collect the essential data points like vehicle density, speed, timing, etc which can be used for traffic prediction and traffic light control. The system mimics real-world road conditions, where continuous vehicle movement can be monitored to provide insights into traffic patterns at junctions or along roads.

**Fig. 12:**
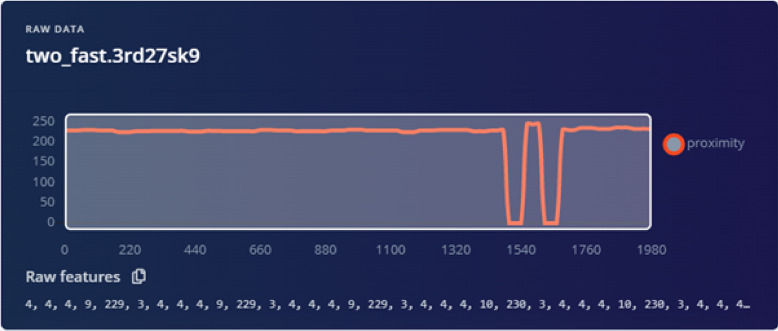
Classification sample raw data. Initial raw data corresponding to the classification sample shown in the previous figure.

The Arduino Nano 33 BLE Sense board is core of the setup, which processes collected data by the sensors in real time. The in-built proximity sensor and BLE is used for traffic simulations and IoT application. The sensors measure the time of arrival and departure of vehicles in the simulation zone, allowing for speed and density calculations. The system is powered by a DC voltage source, and data is processed in real-time and stored for further analysis. This setup ensure efficient and accurate data collection for training and testing the traffic prediction model. Once the traffic data is captured, the Arduino board uses machine learning algorithms to analyze the data and predict traffic behavior. These predictions can be used to optimize traffic light controls, reduce congestion, or adjust road signals based on traffic flow trends. The prototype is a testbed and can be used for smart city solutions for real-time traffic management. The integration of machine learning and real-time data collection is used to make adaptive decisions, such as adjusting traffic light timing or signaling based on predictive analysis. This is a critical development in urban planning, where optimizing traffic flow is essential to reducing congestion and improving overall road efficiency and safety. The sample result is shown in Fig. 12. The figure represents raw proximity data labeled as ”two_fast.3rd27sk9” and shows a nearly steady proximity value around 230, with two significant spikes (dips) occurring between timestamps 1540 and 1760 ms. These spikes indicate sudden changes in proximity, likely caused by rapid or irregular movement of a vehicle detected by the sensors. Such dips could represent instances where the vehicle momentarily moved out of the sensor’s range or a sudden change in distance, simulating either a quick deceleration or an obstacle detection. The rest of the graph shows stable data, suggesting continuous, consistent vehicle movement except for these anomalies.

The collected data sets are divided into two groups for the simulation analysis: a train set for the algorithm training and a test set for the algorithm validation. Seven parameters were created and incorporated into data training and testing sets with proper labeling as shown in Table 2.

For the simulation analysis, the design tasks are divided into 3 process steps, i.e., Live classification, Model Testing, and Deployment.

### Live classification

It enabled the validation of the machine learning models with data captured directly from Arduino Nano 33 BLE Sense. The proposed prototype embedded system communicates via the serial communication mode connected to the Edge impulse account^11^. This connection is established by the command line interface of edge impulse running locally on a nodejs server. This feature provides a way to test the model’s performances in real-time by acquiring data in live and output being processed without offline deployment. This helps us to understand the current capabilities of the model and find out if any adjustments are needed.

### Model testing

**Fig. 13:**
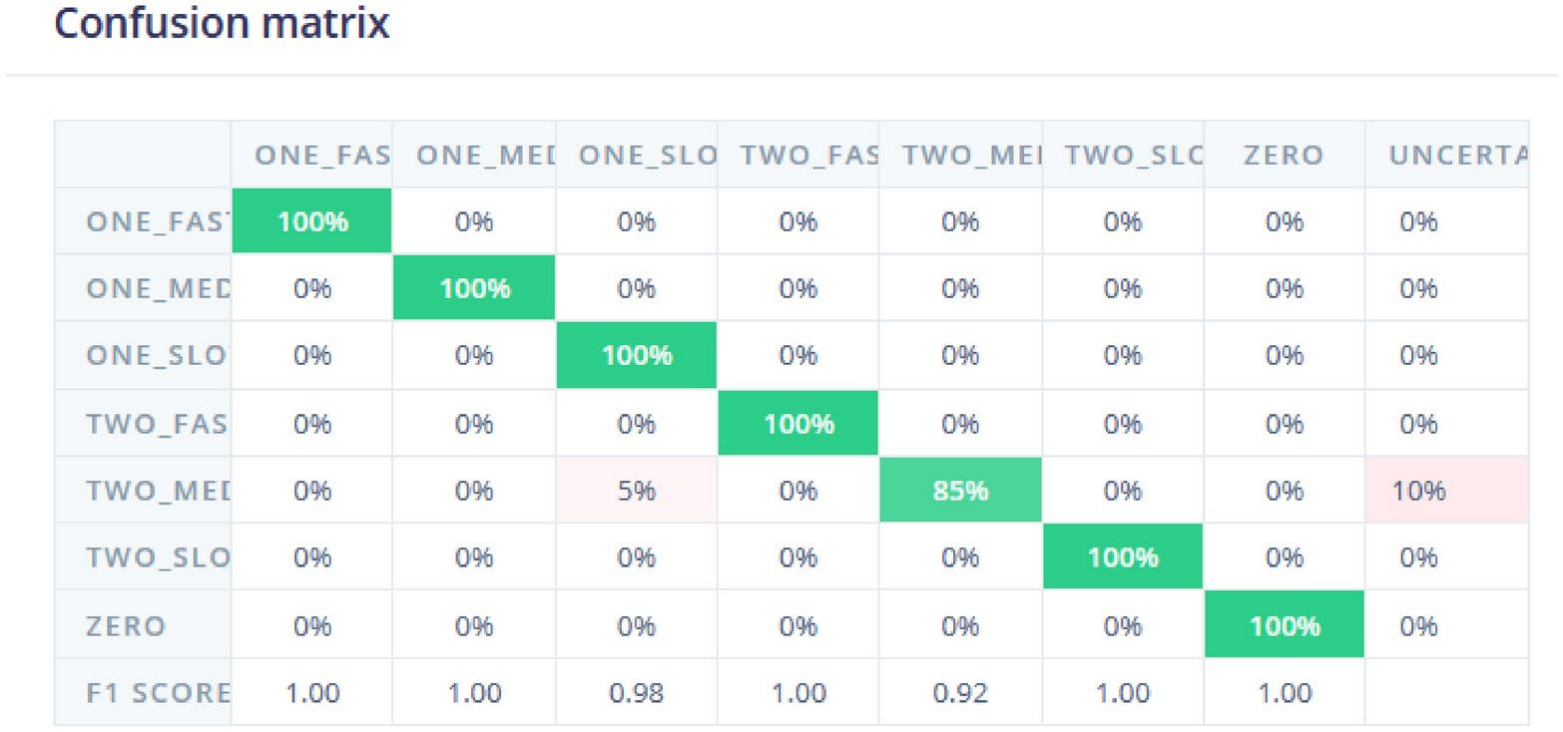
Model Testing Results. Performance metrics and outcomes from testing the predictive model on a test dataset.

The model testing evaluated the model’s performances based on the test dataset. The goal is to assess the model’s accuracy, precision, and other performance metrics, and to identify any issues or areas for improvisation in the model. Model testing is an iterative process that involves adjusting the model’s parameters, such as the learning rate, number of layers, and activation functions, and retesting the model until the desired performance is achieved. This is a critical step in the development of the machine learning model. The sample model testing results are shown in Fig. 13. The classification performance of a model used to predict different traffic speed categories such as ”ONE_FAST,” ”ONE_MED,” ”ONE_SLOW,” ”TWO_FAST,” and others. The matrix shows excellent accuracy for most categories, with 100% correct predictions for ”ONE_FAST,” ”ONE_MED,” ”ONE_SLOW,” ”TWO_FAST,” ”TWO_SLOW,” and ”ZERO.” These green-highlighted cells indicate that the model is highly effective in predicting these classes without any errors. However, some misclassifications occurred in the ”TWO_MED” category, where 5% of instances were incorrectly classified as ”ONE_MED,” and 10% were labeled as ”UNCERTAIN.”

The F1 scores, which measure the balance between precision and recall, are very high across most categories, with values close to 1.00, indicating strong model performance. The only exception is the ”TWO_MED” category, which has a slightly lower F1 score of 0.92 due to misclassifications. Despite this, the overall performance of the model is excellent, but there is room for improvement in refining the classification of the ”TWO_MED” category to further enhance its accuracy.

### Deployment

Edge Impulse^11^ provides built-in deployment targets that allow the creation of ready-to-go binaries for development boards. This feature is involved in directly optimizing and transferring the machine learning model into Arduino Nano 33 BLE.

The deployment process involves optimizing the machine learning model for the target device, which involves quantization, pruning, and other techniques to reduce the model size and improve performance. The optimized model is then compiled into a binary format that can be deployed to the Arduino Nano 33 BLE. Deploying the model to edge devices allows the model to run without an internet connection, minimizing latency, and running with minimal power.

## Result analysis and discussion

The results are represented in multiple formats as shown. The dataset used in this study consists of CBOR-encoded files generated by the Edge Impulse platform, which directly interfaced with the hardware unit to collect real-time data. This hardware unit, equipped with proximity sensors and a microcontroller, detected vehicles and transmitted relevant readings such as speed and density. Edge Impulse encoded the collected real-time data into CBOR format and organized it into traffic categories such as ”zero vehicles,” ”one slow-speed vehicle,” and ”two fast-speed vehicles.” The platform also facilitated preprocessing, including normalization and segmentation into training and testing datasets, ensuring balanced representation across all classes. The training data was used to train the neural network model, while the testing data validated its accuracy and performance. The accuracy of the deployed TinyML model is displayed in the dashboard of the Edge Impulse Studio^11^. Along with the accuracy, the loss is also displayed. The accuracy of 95.5% and Loss of 0.13 is very efficient considering the number of parameters we are dealing with here. With a total of 7 parameters (Labels in Table 2), the predicted output of the TinyML model is highly accurate using the default Keras libraries. On utilizing the Rainforest or Decision Tree algorithms, the accuracy reduced drastically (the details are attached in ’supplementary file’).

The raw data of the two_fast samples are shown in Fig. 12. The results show that there are two spikes. The two spikes represent two vehicles. The speed of vehicles can be predicted based on the inter-distance between spikes (spike width), e.g., low, medium, and fast. The circular motion of the platform allows for continuous vehicle detection, where the rotation speed of the platform can be treated as a scaled representation of high-density traffic scenarios. Proximity sensors are strategically placed to capture data such as vehicle presence, speed, and density by detecting the toy vehicles as they pass specific points. These sensors provide real-time data to a microcontroller (e.g., Arduino Nano), which processes and transmits the information for further analysis. By simulating varying rotation speeds, the prototype effectively models different traffic densities, allowing for scaled testing of high-density scenarios. This setup ensures a scalable and adaptable approach for validating traffic prediction systems, with a simplified yet effective representation of real-world traffic patterns.

The confusion matrix is plotted in Fig. 10 with the input labels as the rows and predicted labels as the columns. The confusion matrix’s cells each denote the ratio of No. of Predicted labels which are correct to the Total no. of input samples of the same label. In the Edge Impulse Studio^11^, We find the values to be percentages which denote the accuracy of the Predicted label to the input label. The highest inaccurate prediction was made for the one_slow labeled inputs. 14.7% of the times, the model would predict one_slow as two_fast is what can be inferred from the confusion matrix. The data explorer shows how the neural network classifies each label. The greener the color, more accurate will be the result of the model deployed.

**Fig. 14:**
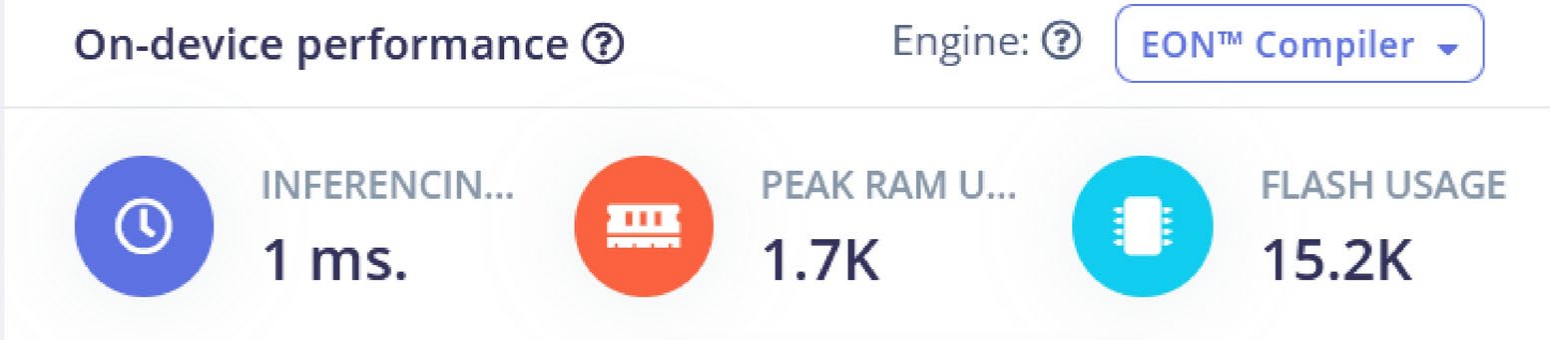
Inferencing time, peak time and flash usage. Performance analysis in terms of inferencing time, peak processing time, and flash memory usage.

The Inferencing time is shown in Fig. 14 along with the Peak RAM usage and Flash Usage. These statistics help in finding the right target for the model to be deployed onto. The on-device performance metrics show that the machine learning model is highly efficient, with a rapid inference time of 1 millisecond, peak RAM usage of just 1.7K, and flash usage of 15.2K. These metrics suggest the model is optimized for real-time applications and can run effectively on resource-constrained devices, making it suitable for low-power, embedded systems such as traffic control or IoT devices.

**Fig. 15:**
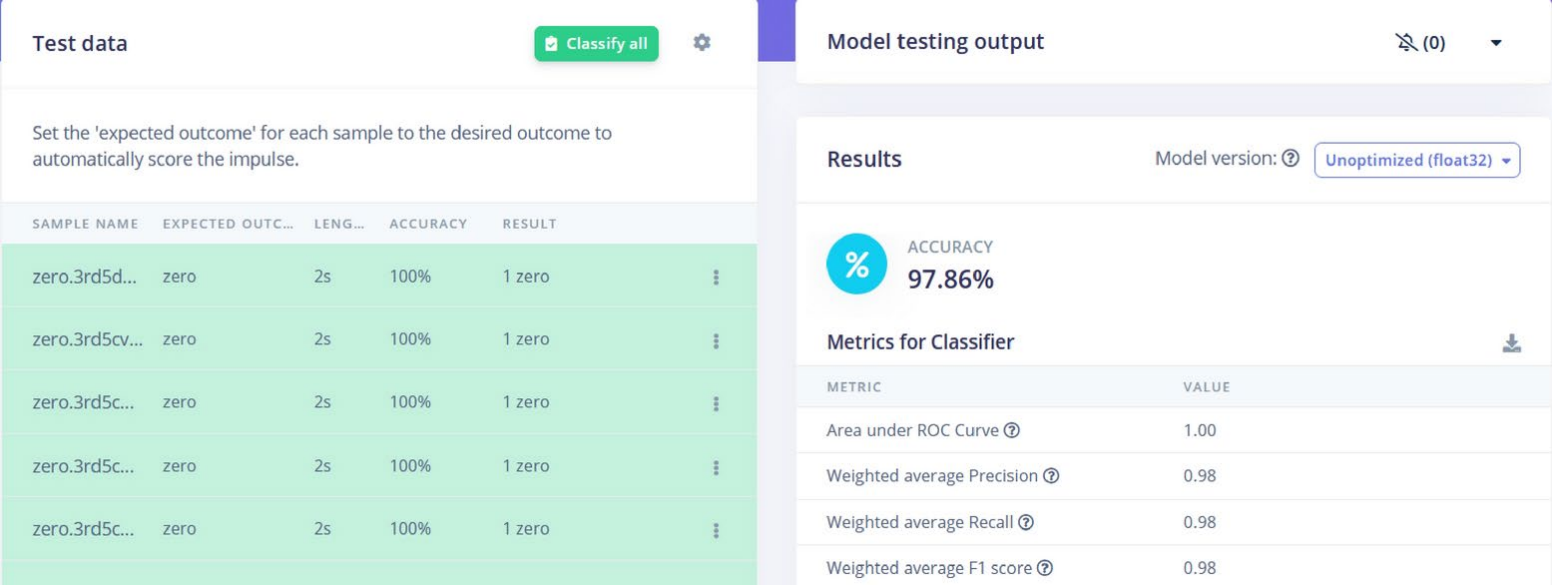
Result analysis of testing data. Detailed examination and comparison of predicted versus actual results from the test data.

The model is further tested with live samples as shown in Figs. 13 and 15. The validation metrics for classification are derived from the confusion matrix. These metrics provide a comprehensive evaluation of the model’s classification performance. As per the result, the model achieves an accuracy of **97.86%**, which indicates that almost all classifications were correct. Accuracy measures the proportion of correctly classified samples out of the total samples. It is mathematically defined as:

1 $$\begin{aligned} \text {Accuracy} = \frac{\text {Sum of Diagonal Elements}}{\text {Total Number of Samples}} \end{aligned}$$

Here, the diagonal elements in the confusion matrix represent correctly classified samples for each class. Figure 13 shows the confusion matrix provides a detailed breakdown of the model’s predictions for each class. The matrix shows perfect classification (100%) for classes like *ONE_FAST*, *ONE_SLC*, and *ZERO*. For the *TWO_MED* class, the model shows minor misclassification with a small percentage of predictions falling into the *TWO_SLOW* class. This matrix is essential for identifying specific areas where the model performs well or needs improvement.

The F1-score is also analyzed which is harmonic mean of precision and recall, balancing their contributions to evaluate the model’s performance. For instance, the figure reports an F1-score of **1.00** for several classes, indicating perfect precision and recall for those categories. It is mathematically defined as:

2 $$\begin{aligned} \text {F1-Score} = 2 \times \frac{\text {Precision} \times \text {Recall}}{\text {Precision} + \text {Recall}} \end{aligned}$$

**Fig. 16:**
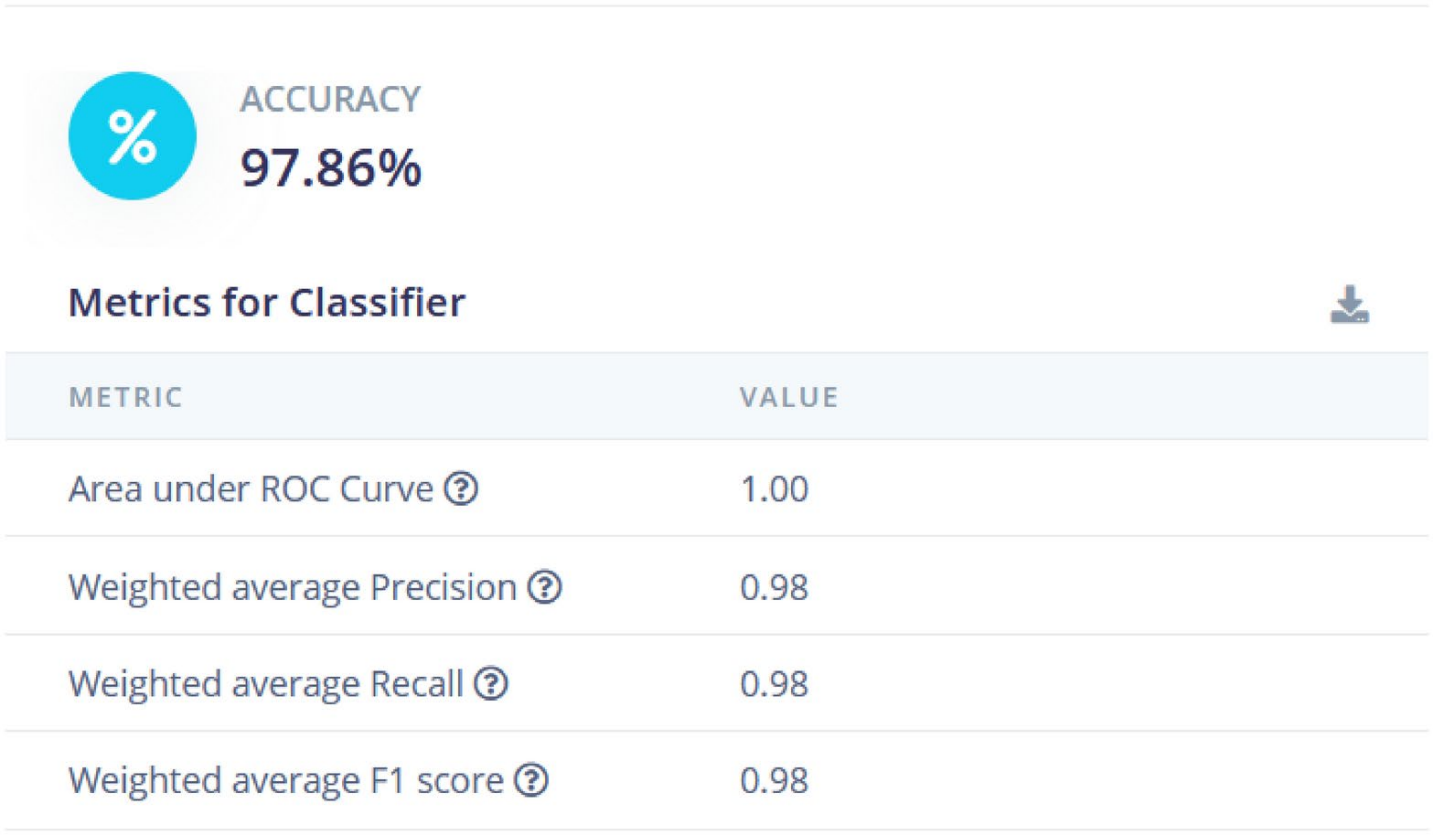
Classification results and metrics. Example output showcasing the classification results of a data sample by the model.

These metrics, derived directly from the confusion matrix and associated statistics, demonstrate the model’s strong performance across most traffic categories, while highlighting areas for minor improvement. For the collection of the data, we have generated different data samples for different scenarios, e.g., one_slow, one_fast. The results are collected in the separate files for each scenario as shown in Fig. 16. The metrics for the classifier include an Area Under the ROC Curve (AUC) of 1.00, indicating excellent model performance in distinguishing between classes. The weighted average precision, recall, and F1 score are all at 0.98, showing that the model has a well-balanced performance across different metrics. These results indicate that the model is highly reliable for traffic classification, though minor refinements may be needed to further enhance its accuracy.

**Fig. 17:**
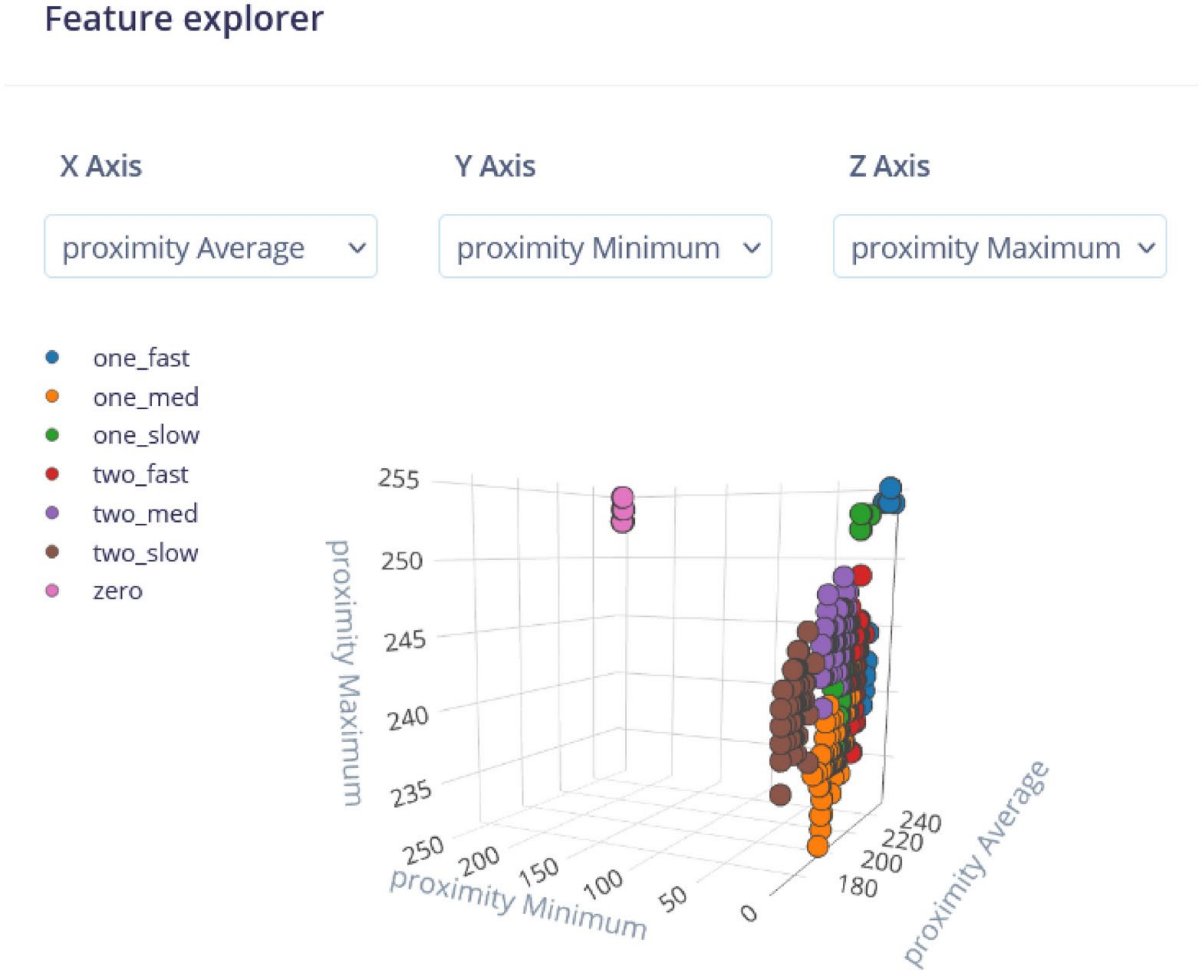
Flatten X,Y, and Z axis. Transformation of the three-dimensional data into a flattened format for easier analysis.

**Fig. 18:**
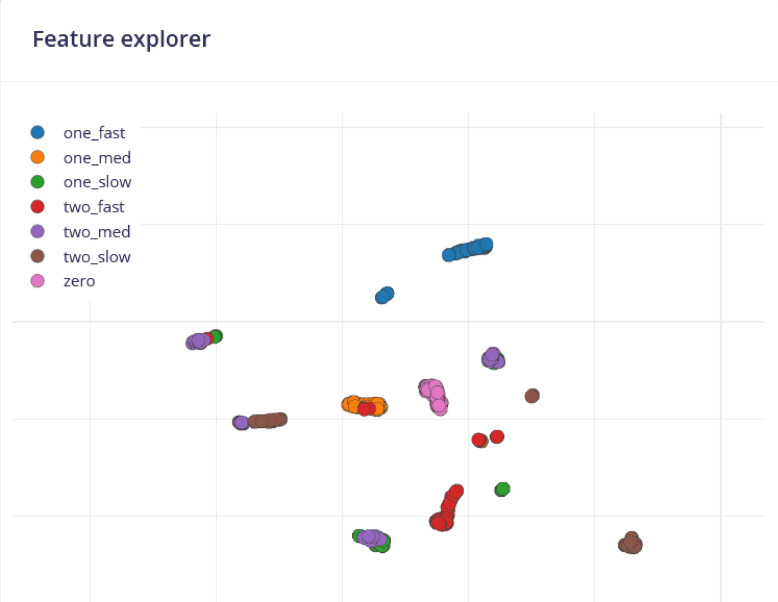
Spectral Features. Extracted spectral features from the processed data used for enhancing model accuracy.

**Table 3:** Comparative analysis of the different ML methods.

ML Method	Accuracy
Random Forest Algorithm	54%
Logistic Regression	28%
K-Nearest Neighbour	51.3%
Decision Tree	53.5%
Proposed Model	97.86%

The results are also analyzed in terms of flatten processing block as shown in Fig. 17. The flatten processing block allows us to observe the samples as entities in a 3-dimensional plot. The axis of this plot are X - Proximity Average, Y - Proximity Minimum and Z - Proximity Maximum. These features can be replaced with Proximity RMS, Skewness, etc. This enables to comprehend the analog signals and samples in convenient visual representation. The results show that the proximity-based features (Average, Minimum, Maximum) provide good separation between most traffic classes, as evidenced by the distinct clusters for classes like ”zero” and ”one_fast.” This suggests that these features are effective in distinguishing different traffic patterns. However, some overlap is observed between certain classes, such as ”two_fast” and ”two_med,” which indicate similarities in their proximity values. The overlap could potentially lead to classification errors and suggests a need for further feature refinement or additional features to better separate these classes. The proximity features are useful, but some classes may benefit from additional analysis to improve distinction.

The samples of the spectral features are shown in Fig. 18. The samples can be easily classified in the graph based on labels of different traffic. There is some overlap between other classes, such as ”one_med” (orange) and ”two_med” (brown), suggesting that these classes share similar feature values and may be harder for the model to distinguish.

To validate the analysis, the proposed method is analyzed experimentally for small-scale traffic conditions. The prototype is utilized in our college entrance, where the traffic was observable and predictable. We have monitored real-world traffic but on a small scale of college entrance. The traffic used to be around 360 two tired vehicles, and 15 four-wheeled vehicles. The study was conducted at a distance of 100 meters from the college entrance point. The prediction of traffic at the entrance was successful when there were no interventions of people walking around.

The performance of the proposed method is compared with the existing conventional ML methods. The results are shown in Table 3. The details of the comparative analysis are given in ’Supplementary Material’. The results prove the superiority of the proposed method.

## Conclusion

This paper proposes and designs a traffic prediction and control system using TinyML, implemented with Edge Impulse Studio and Arduino IDE. The prediction model has been optimized to achieve 97.86% accuracy by considering all 7 parameters of traffic and predicting when the vehicles reach the junction. Arduino Nano 33 BLE Sense board is used as our detector of traffic. Since its proximity sensor is designed for short range, we built a prototype depicting real-time traffic and recorded samples using this prototype. We created an interface between our sensor and Edge impulse platform to record samples and split these into 80% and 20% to training data and testing data respectively. The proposed model can be a potential alternative to adaptive traffic signals that exist in major cities of our country.

In the future the proposed model will be analyzed for high-density traffic and will be deployed in the field for real-time analysis. The primary limitation of the proposed method is its optimization based on the traffic environment, as traffic conditions vary across different junctions. Additionally, the proposed work has been analyzed only for one-way traffic, which may limit its applicability in more complex multi-directional traffic scenarios.

## Supplementary Information

Supplementary Information.

## Data Availability

The dataset generated during this study is available upon reasonable request. Interested researchers or authors may contact the corresponding author, Manoj Tolani, at manoj.tolani@manipal.edu.

## Abbreviations

TCS: Traffic control system
ML: Machine learning
TinyML: Tiny machine learning
MLP-NN: Multi-layer perceptron neural network

## Materials availability

Materials used in this study are available from the corresponding author on reasonable request. The interested researcher/author can contact to corresponding author on manoj.tolani@manipal.edu email id.
