# Supplementary material for: Machine learning based adaptive traffic prediction and control using edge impulse platform
Source: Sci Rep. 2025 May 17;15:17161. doi: 10.1038/s41598-025-00762-4 (PMC12085571; doi:10.1038/s41598-025-00762-4)
Supplement: Supplementary file 1 — Supplementary Information. [file 41598_2025_762_MOESM1_ESM.pdf]

Supplementary Material

Code

```
import tensorflow as tf

from tensorflow.keras.models import Sequential

from tensorflow.keras.layers import Dense, InputLayer, Dropout, Conv1D, Conv2D, Flatten, Reshape, MaxPooling1D, MaxPooling2D, AveragePooling2D, BatchNormalization, Permute, ReLU, Softmax

from tensorflow.keras.optimizers.legacy import Adam

EPOCHS = args.epochs or 1700

LEARNING_RATE = args.learning_rate or 0.0005

# this controls the batch size, or you can manipulate the tf.data.Dataset objects yourself

BATCH_SIZE = 32

train_dataset = train_dataset.batch(BATCH_SIZE, drop_remainder=False)

validation_dataset = validation_dataset.batch(BATCH_SIZE, drop_remainder=False)

# model architecture

model = Sequential()

model.add(Dense(20, activation='relu',

    activity_regularizer=tf.keras.regularizers.l1(0.00001)))

model.add(Dense(10, activation='relu',

    activity_regularizer=tf.keras.regularizers.l1(0.00001)))

model.add(Dense(classes, name='y_pred', activation='softmax'))

# this controls the learning rate

opt = Adam(learning_rate=LEARNING_RATE, beta_1=0.9, beta_2=0.999)

callbacks.append(BatchLoggerCallback(BATCH_SIZE, train_sample_count, epochs=EPOCHS))

# train the neural network

model.compile(loss='categorical_crossentropy', optimizer=opt, metrics=['accuracy'])

model.fit(train_dataset, epochs=EPOCHS, validation_data=validation_dataset, verbose=2, callbacks=callbacks)

# Use this flag to disable per-channel quantization for a model.

# This can reduce RAM usage for convolutional models, but may have

# an impact on accuracy.

disable_per_channel_quantization = False
```

Comparative Analysis of Various Machine Learning Algorithms

Random Forest Algorithm output:

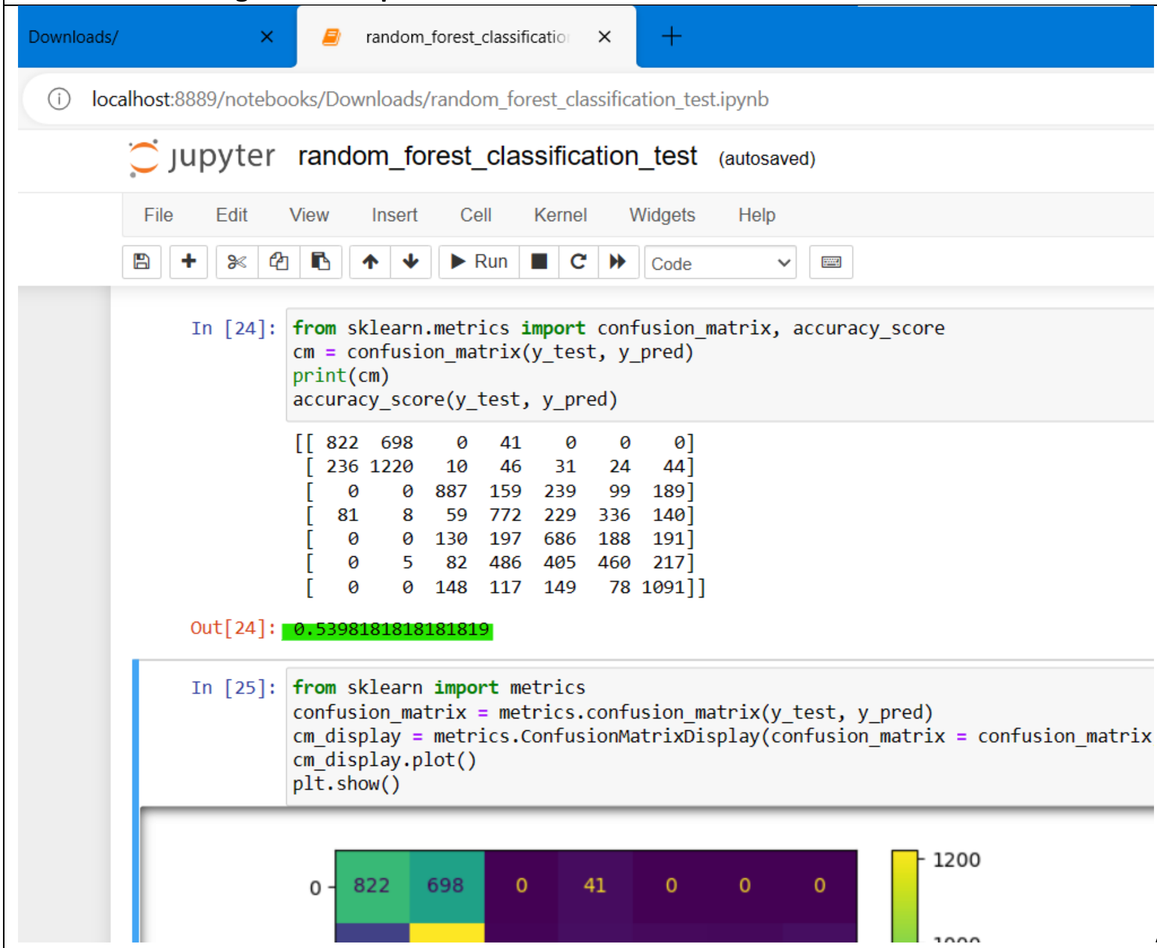

Accuracy is ~53%

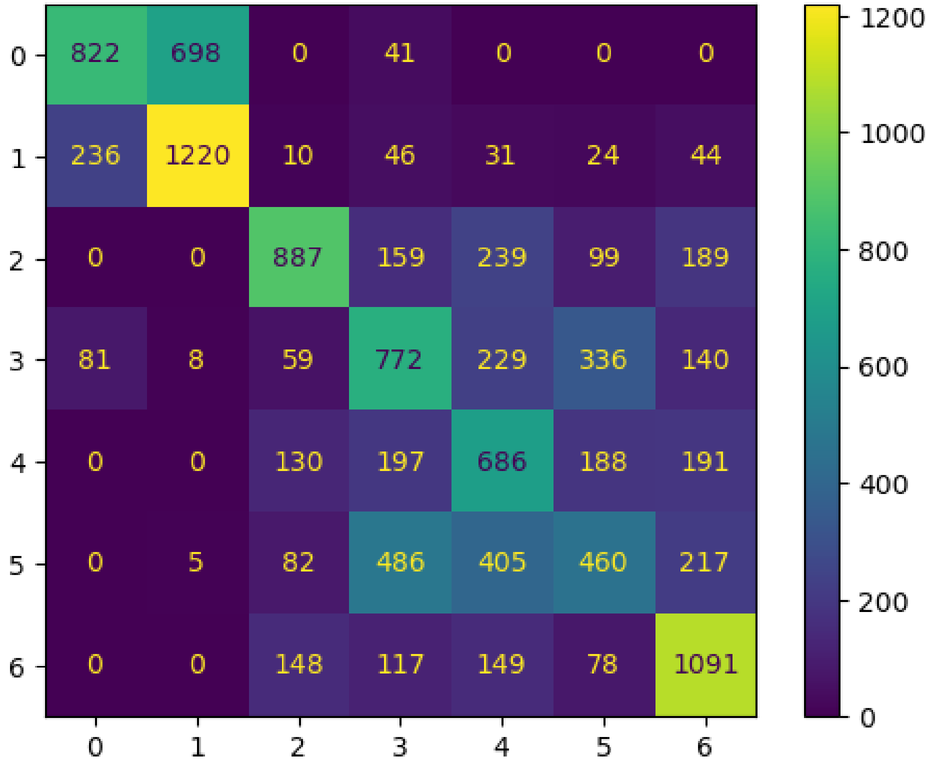

Logistic Regression:

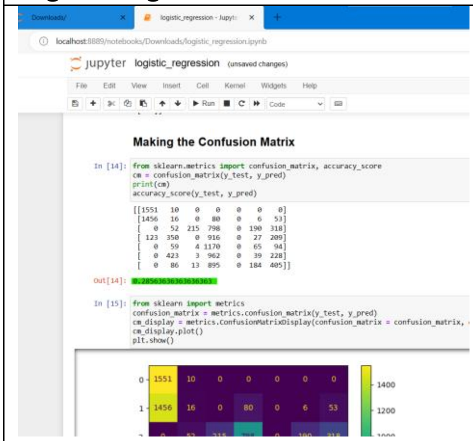

Accuracy ~28%

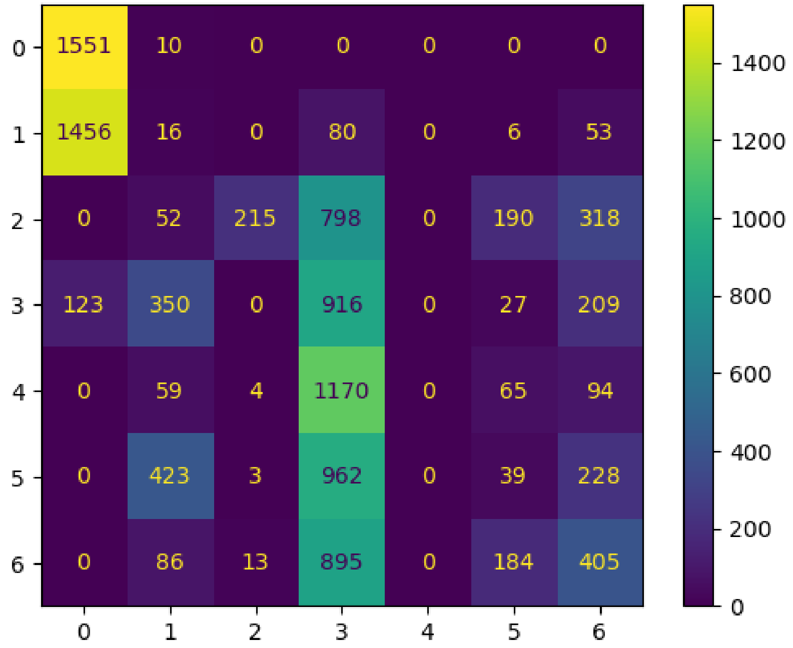

K-Nearest-Neighbors:

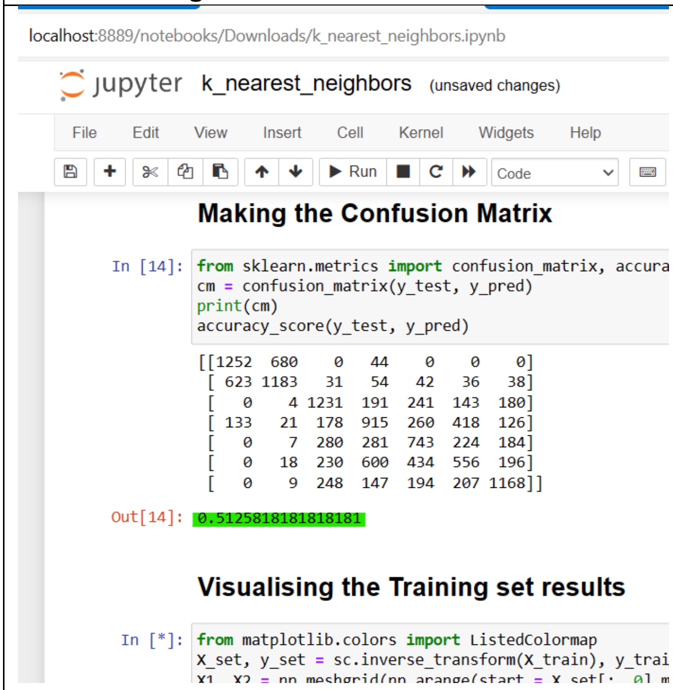

Accuracy ~51.3

Decision tree algorithm:

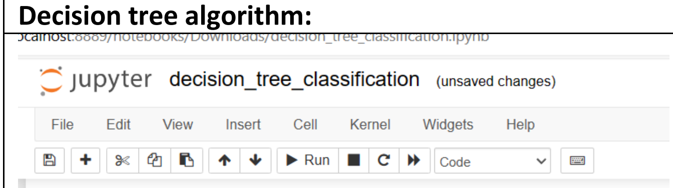

Making the Confusion Matrix

In [14]:
```
from sklearn.metrics import confusion_matrix, accuracy_score
cm = confusion_matrix(y_test, y_pred)
print(cm)
accuracy_score(y_test, y_pred)
```

```
[[1056  877    0   43    0    0    0]
 [ 345 1463   14   65   46   25   49]
 [   0    1 1144  216  290   90  249]
 [ 121    9   93 1002  291  381  154]
 [   0    0  207  254  860  192  206]
 [   0    6  129  664  510  488  237]
 [   0    1  184  166  197   85 1340]]
```

Out[14]: 0.5347636363636363

Accuracy ~53.5
